# Activation of SIRT1 Reduces Renal Tubular Epithelial Cells Fibrosis in Hypoxia Through SIRT1‐FoxO1‐FoxO3‐Autophagy Pathway

**DOI:** 10.1002/adbi.202400583

**Published:** 2025-04-08

**Authors:** Guangyu Wang, Lijuan Zhang, Jiaorong Tan, Fei Li, Yishan Jin, Limei He, Xin Yang

**Affiliations:** ^1^ Department of Endocrinology Putuo People's Hospital of Tongji University School of Medicine Tongji University Shanghai 200060 China; ^2^ Department of Clinical Laboratory Changning Maternity and Infant Health Hospital East China Normal University Shanghai 200050 China

**Keywords:** autophagy, fibrosis, foxO1, heart failure, hypoxia, SIRT1

## Abstract

Heart failure‐induced renal tubular epithelial cell fibrosis is an important pathological process that leads to chronic kidney disease. This study is to investigate the regulatory mechanism of over‐expression or knock‐down SIRT1 gene, alleviating hypoxia‐induced HK2 cell fibrosis in heart failure. The focus is on the SIRT1‐FoxO1‐FoxO3‐Autophagy pathway. In vitro experiments are performed by HK2cell line to simulate the normal oxygen state (Normoxia) and the hypoxia state (Hypoxia) caused by heart failure, SIRT1 gene over‐expression by transfected vectors, knock‐down and Rapamycin (RAPA)‐induced cellular autophagy, and the cell models are divided into four subgroups, named control group, oeSIRT1, siSIRT1 and siSIRT1+RAPA. Western blotting (WB), real‐time qPCR, immunofluorescence (IF), ELISA, and transmission electron microscopy are used to quantitatively or semi‐quantitatively analyze the expression of FoxO1, FoxO3, SIRT1, Beclin1, LC‐3, α‐SMA, E‐ Cadherin, and collagen‐I in cells or supernatants. It is demonstrated that activation of SIRT1 regulates the expression and activity of FoxO1 and FoxO3, thereby affecting autophagy. This modulation leads to a reduction in HK2 fibrosis markers (α‐SMA and E‐cadherin) and extracellular matrix deposition (collagen I), which ultimately attenuates renal tubular epithelial cell fibrosis. These findings provide new insights into potential therapeutic strategies for treating heart failure‐induced renal tubular epithelial cell fibrosis by targeting the SIRT1‐FoxO1‐FoxO3‐Autophagy pathway.

## Graphical abstract

Hypoxia promotes the epithelial‐mesenchymal transition (EMT) of renal tubular epithelial cells via the SIRT1‐FoxO1‐FoxO3‐autophagy pathway, thereby resulting in the fibrosis of renal tubular epithelial cells. Activation of SIRT1 or induction of autophagy inhibits this process, alleviating hypoxia‐induced fibrosis.

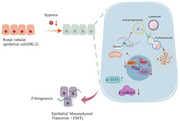

## 1 Introduction

Heart failure is a serious cardiovascular disease that adversely affects renal function during the progression of the disease. In recent years, the incidence and prevalence of heart failure have continued to increase globally, especially in the elderly population and patients with underlying diseases such as myocardial infarction, diabetes mellitus, hyperlipidemia, and hypertension.^[^ ^1^, ^2^ ^]^ Heart failure seriously affects the quality of life of patients, often leading to dyspnea, fatigue, decreased exercise tolerance, and impaired function of the kidneys and other organs.^[^ ^3^ ^]^ Simultaneously, heart failure significantly increases the risk of hospitalization and mortality in patients, placing a heavy burden on healthcare systems. Furthermore, early diagnosis and intervention for heart failure is crucial. The early identification of high‐risk individuals and effective therapeutic measures, such as medication and lifestyle modifications, can delay disease progression and improve survival and quality of life.^[^ ^4^ ^]^

Cardiorenal syndrome (CRS) represents a pathophysiological disorder defined by a bidirectional interplay between cardiac and renal dysfunction. The current classification system delineates five distinct CRS subtypes. Type 1 CRS (acute cardiorenal syndrome) is characterized by acute decompensated heart failure precipitating acute kidney injury, whereas Type 2 CRS (chronic cardiorenal syndrome) manifests as chronic heart failure leading to progressive chronic kidney disease. Both subtypes demonstrate the pathophysiological cascade whereby impaired cardiac function initiates and accelerates renal deterioration. Notably, clinical evidence reveals that heart failure frequently coexists with CRS, and their reciprocal dysfunction establishes a vicious cycle that synergistically exacerbates multiorgan functional decline.^[^ ^5^, ^6^ ^]^ In heart failure, the pumping function of the heart is insufficient, which leads to the restriction of systemic blood circulation and insufficient blood supply to organs, especially the kidneys are most seriously involved, which causes insufficient oxygen supply and this hypoxic state produces direct damage to Renal Tubular Epithelial Cells (RTECs).^[^ ^7^ ^]^ Studies have shown that activation of Hypoxia‐Inducible Factors (HIFs) plays a key role in this pathological process.^[^ ^8^ ^]^ Accumulation of HIFs triggers a series of cellular stress responses, including upregulation of fibrosis‐related genes (e.g., E‐cadherin, N‐cadherin, vimentin, and α‐SMA). Sustained hypoxic stimulation induces phenotypic transformation of RTECs, which gradually lose their normal epithelial cell characteristics and shift to a fibroblast‐like phenotype.^[^ ^9^ ^]^ This transformation process is accompanied by the excessive synthesis and deposition of components such as collagen and fibronectin in the Extracellular Matrix (ECM).^[^ ^9^ ^]^ Over time, the accumulation of ECM in RTECs eventually leads to the development of fibrosis in the renal tubular epithelial cells. This fibrotic alteration of epithelial cells disrupts the normal structure and function of the renal tubule, rendering it unable to efficiently reabsorb and secrete substances. Eventually, extensive fibrosis of the renal tubular epithelial cells progresses, leading to renal failure, which seriously affects the survival and prognosis of patients.^[^ ^7^ ^]^

Sirtuins (SIRTs) are a class of NAD+‐dependent histone deacetylases that are evolutionarily conserved, involved in many physiological processes, and regulate a range of key signaling pathways. There are seven recognized members of the SIRT family, SIRT1–SIRT7. The SIRT protein family binds to proteins such as p53, FOXO/PGC‐1α, NF‐κB, Ku70, and others, and regulates important processes such as cellular stress response, metabolism, senescence, and apoptosis.^[^ ^10^, ^11^ ^]^ At present, there is a large body of evidence that the SIRT protein family is widely involved in the development of tumors, cardiovascular and cerebrovascular diseases, respiratory diseases, digestive diseases, and other diseases.^[^ ^12^, ^13^, ^14^ ^]^ Therefore, the SIRT protein family is valuable for medical research and clinical practice.

FoxO1 is a member of the forkhead box (Fox) family of transcription factors that play an important role in cell metabolism, stress response, and apoptosis. SIRT1 regulates transcriptional activity by deacetylating FoxO1. Cellular autophagy is an intracellular degradation process that removes damaged organelles and proteins and maintains the stability of the intracellular environment by forming autophagosomes and fusing them with lysosomes. Studies have shown that FoxO1 is involved in regulating the expression of autophagy‐related genes and promoting cellular autophagy, thereby maintaining homeostasis in the tissue environment.^[^ ^11^ ^]^ During heart failure‐induced renal tubular fibrosis, the level of cellular autophagy is reduced, leading to the accumulation of damaged organelles and proteins, which exacerbates cellular damage and further fibrosis of epithelial cells, and activation of the SIRT1‐FoxO1 signaling pathway promotes autophagy, which attenuates cellular damage and fibrosis.^[^ ^11^ ^]^ These studies suggest that understanding the pathogenic mechanisms of heart failure complicated by renal tubular epithelial cell fibrosis (TECF) is of great clinical value for developing new therapeutic approaches and improving patient prognoses.

In our previous study, we conducted long‐term observational studies on patients with clinical heart failure and renal dysfunction and explored this synchronously in a mouse model of cardiorenal syndrome (not published). The aim of this study was to investigate the mechanisms between heart failure‐induced renal fibrosis and the SIRT1‐FoxO1‐FoxO3‐autophagy pathway within the framework of cardiorenal syndrome. Thus, we hypothesized that heart failure may regulate the pathological process of renal tubular epithelial cell fibrosis through the SIRT1 protein and explored the mechanism of heart failure‐induced renal tubular epithelial cell fibrosis by constructing HK2 a Human kidney cell line) cell culture system and simulating the hypoxic environment caused by heart failure in vitro. By constructing HK2 a Human kidney cell line) cell culture system in vitro, simulated the hypoxic environment caused by heart failure and investigated the mechanism of heart failure‐induced tubular epithelial cell fibrosis, which can help us understand the pathophysiological mechanism of heart failure‐induced tubular fibrosis and has important value for the prevention and treatment of renal tubular fibrosis.

## 2 Results

### 2.1 Hypoxia‐Induced Down‐Regulation of SIRT1‐FoxO1‐FoxQ3 Expression Levels in HK2 Cells in Contrast to Normoxia

**Figure 1:**
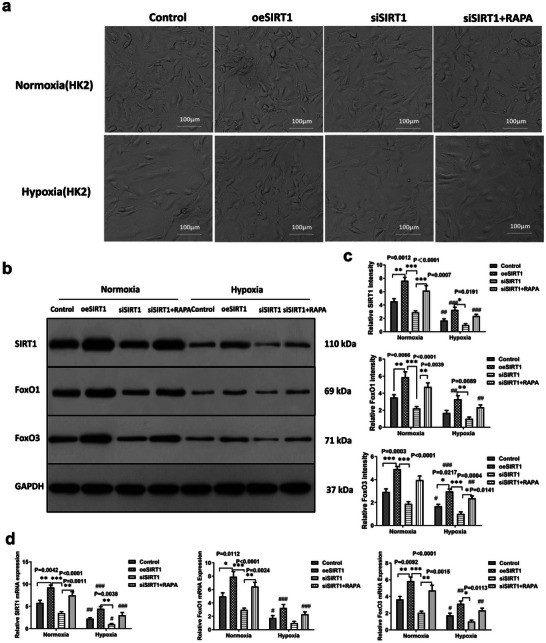
Effects of over‐expression, knock‐down of SIRT1 and induction of autophagy on HK2 cell growth. a) Morphology of HK2 cells in the Control, oeSIRT1, siSIRT1, and siSIRT1+RAPA groups was observed. In the Control group, cells displayed typical morphology with uniform size and shape. In the oeSIRT1 group, cells showed increased density and altered shape. In the siSIRT1 group, cells appeared smaller and less organized. In the siSIRT1+RAPA group, cells presented a distinct morphology compared to the siSIRT1 group. (*n* = 3 samples per group). b) Western blot analysis was performed to detect the expression levels of SIRT1, FoxO1, FoxO3, and GAPDH (used as a normalization gene) under normoxia and hypoxia conditions. (*n* = 3 samples per group, Control versus oeSIRT1 versus siSIRT1 versus SIRT1+RAPA: ^*^ *p* < 0.05, ^**^ *p* < 0.01, ^***^ *p* < 0.001. Group under normoxia versus hypoxia: ^#^ *p* < 0.05, ^##^ *p* < 0.01, ^###^ *p* < 0.001). c) The relative intensity of SIRT1, FoxO1, and FoxO3 compared to the Control group was determined by western blot analysis (The Discovery Series Quantity One 1‐D Analysis Software Version 4.6.8, Bio‐Rad, USA). (*n* = 3 samples per group, Control versus oeSIRT1 versus siSIRT1 versus SIRT1+RAPA: ^*^ *p* < 0.05, ^**^ *p* < 0.01, ^***^ *p* < 0.001. Group under normoxia versus hypoxia: ^#^ *p* < 0.05, ^##^ *p* < 0.01, and ^###^ *p* < 0.001). d) The relative mRNA expression levels of SIRT1, FoxO1, and FoxO3 were evaluated. (*n* = 3 samples per group, Control versus oeSIRT1 versus siSIRT1 versus SIRT1+RAPA: ^*^ *p* < 0.05, ^**^ *p* < 0.01, and ^***^ *p* < 0.001. Group under normoxia versus hypoxia: ^#^ *p* <0.05, ^##^ *p* < 0.01, and ^###^ *p* < 0.001). All experiments were repeated three times independently of each other. Note: Control versus oeSIRT1 versus siSIRT1 versus SIRT1+RAPA: ^*^ *p* < 0.05, ^**^ *p* < 0.01, and ^***^ *p* < 0.001. Group under normoxia versus hypoxia: ^#^ *p* < 0.05, ^##^ *p* < 0.01, and ^###^ *p* < 0.001. oeSIRT: overexpression of SIRT; siSIRTI: SIRTI knockdown using siRNA; siSIRTI+RAPA: knockdown of SIRT1 using siRNA with rapamycin‐induced autophagy.

We divided HK2 cells into four groups for culture under both normoxia and hypoxia conditions, constructed vectors over‐expression of SIRT1 (oeSIRT1 group), knockdown of SIRT1 (siSIRT1 group); set up RAPA‐induced autophagy in siSIRT1 group, and observed the morphological changes of HK‐2 cells in each group under inverted light microscope; morphological changes of HK‐2 cells in each group under both normoxia and hypoxia conditions. Under normoxic conditions, HK2 cells cultured in the control group grew adherently to the wall, showing a paving stone‐like morphology with tightly arranged and ordered cells. The cells in the hypoxia group were sparse, showing an enlarged and deformed status; the oeSIRT1 group showed significant changes in cell morphology and number, the number of cells in the siSIRT1 group was small, the morphology was large, the number of cells in the siSIRT1+RAPA group was increased compared with that in the siSIRT1 group, and the cell morphology was regular (**Figure** **1**a).

After culturing the eight groups of cells for 48 h, western blotting and Real‐time‐q PCR were performed to detect SIRT1, FoxO1, and FoxO3. The results showed that the expression of oeSIRT1 was increased in the SIRT1 group compared to that in the control group under normoxic conditions, and the expression of SIRT1, FoxO1, and FoxO3 was significantly decreased in the siSIRT1 group compared to that in the oeSIRT1 group. SIRT1 expression was increased after RAPA‐induced autophagy (Figure 1c,d, *star sign and *p* value are shown in figures). Compared with normoxia, control, oeSIRT1, and siSIRT1+RAPA expression of SIRT1 were significantly decreased under Hypoxia (Figure 1c upper and Figure 1d left, #hash sign shows sig. The *p* values of WB were *p* = 0.0012, 0.0001, 0.0654, and 0.001, respectively; the *p* values of real‐time PCR were *p* = 0.0032, 0.001, 0.0464, and 0.003). Compared with normoxia, oeSIRT1 and siSIRT1+RAPA expression of FoxO1 were significantly decreased in Hypoxia (Figure 1c middle and Figure 1d middle, #hash sign shows sig., The *p* values of WB were respectively 0.0511, 0.0031, 0.3422, and 0.0066; The *p* values of real‐time PCR were respectively 0.0052, 0.001, 0.1601, and 0.004). Compared to Normoxia, in Hypoxia, oeSIRT1 and siSIRT1+RAPA expression of FoxO1 was significantly decreased (Figure 1c middle and Figure 1d middle, #(hash) sign shows sig. The *p* values of FoxO3 WB for *p* = 0.0281, 0.0004, 0.1955, and 0.0141; the *p* values of real‐time PCR were 0.0278, 0.0013, 0.4557, and 0.0044). Interestingly, the induced autophagy group (siSIRT1+RAPA) showed elevated expression of SIRT1, FoxO1, and FoxO3 (Figure 1b–d).

### 2.2 Hypoxia‐Induced Levels of Autophagy Reducing in HK2 Cells Compare with Normoixa Condition, Leading to HK‐2 Cell Shown Fibrosis

**Figure 2:**
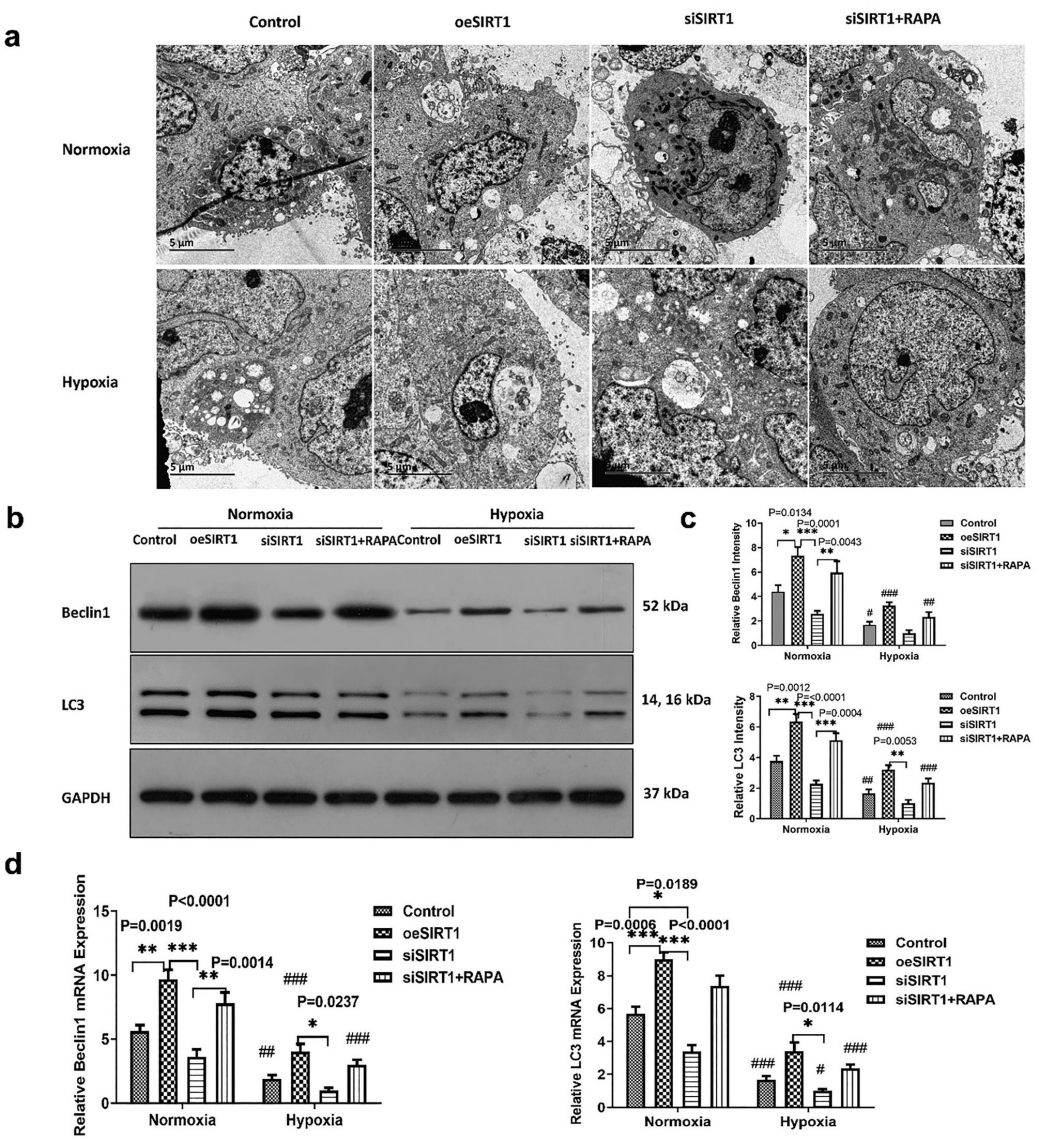
The effect of knock‐down, over‐expression of SIRT1, and autophagy inducing by RAPA on HK2 cell autophagy (*n* = 3), All experiments were repeated three times independently of each other. a) Autophagosome of HK2 cell in the Control, oeSIRT1, siSIRT1, and siSIRT1+RAPA groups was observed using transmission electron microscope(TEM) Images. b) Western blot analysis was performed to detect the expression levels of Beclin1, LC3, and GAPDH (used as a normalization gene) under normoxia and hypoxia conditions. c) The relative intensity of Beclin1 and LC3 compared to the Control group was determined by western blot analysis software(The Discovery Series Quantity One 1‐D Analysis Software Version 4.6.8, Bio‐Rad, USA). (*n* = 3 samples per group, Control versus oeSIRT1 versus siSIRT1 versus SIRT1+RAPA: ^*^ *p* < 0.05, ^**^ *p* < 0.01, and ^***^ *p* < 0.001.Group under normoxia versus hypoxia: ^#^ *p* < 0.05, ^##^ *p* < 0.01, and ^###^ *p* < 0.001). d) The relative mRNA expression levels of Beclin1 and LC3 were evaluated using real‐time‐q‐ PCR. (*n* = 3 samples per group, Control versus oeSIRT1 versus siSIRT1 versus SIRT1+RAPA: ^*^ *p* < 0.05, ^**^ *p* < 0.01, and ^***^ *p* < 0.001.Group under normoxia versus hypoxia: ^#^ *p* < 0.05, ^##^ *p* < 0.01, and ^###^ *p* < 0.001). Note: Control versus oeSIRT1 versus siSIRT1 versus SIRT1+RAPA: ^*^ *p* < 0.05, ^**^ *p* < 0.01, and ^***^ *p* < 0.001. Group under normoxia versus hypoxia: ^#^ *p* < 0.05, ^##^ *p* < 0.01, and ^###^ *p* < 0.001. oeSIRT: overexpression of SIRT; siSIRTI: SIRTI knockdown using siRNA; siSIRTI+RAPA: knockdown of SIRT1 using siRNA with rapamycin‐induced autophagy.

To explore autophagy under hypoxia, we used electron microscopy to examine each group of cells, which showed that the number of autophagic vesicles increased in the SIRT overexpression group (oeSIRT1) compared to the control group, the number of autophagic vesicles decreased in the SIRT1 knockdown group (siSIRT1) compared to the control group, and autophagy was induced in the SIRT1 knockdown combined with RAPA group (siSIRT1+RAPA) compared to the siSIRT1 group (**Figure** **2**a).

We further examined the autophagy markers Beclin1 and LC3, and the results showed that oeSIRT1 was increased in the Beclin1 group compared to the control group under normoxic conditions, and the expression of Beclin1 and LC3 was significantly decreased in the siSIRT1 group compared to that in the oeSIRT1 group. After RAPA‐induced autophagy, the expression of Beclin1 and LC3 was elevated (Figure 2b–d, star sign and *p* value were observed; * the star sign and *p*‐value are shown significantly in the figures).

Beclin1 expression was significantly decreased in the control, oeSIRT1, and siSIRT1+RAPA in Hypoxia compared to normoxia (Figure 2c upper and Figure 2d left, #(hash) sign shows significantly (sig.). The *p* values of WB were respectively 0.0260, 0.0007, 0.3918, and 0.0023; The *p* values of RT‐PCR were respectively 0.0045 < 0.0001, 0.0653, and 0.0003); compared with normoxia, in Hypoxia, the oeSIRT1 and siSIRT1+RAPA expression of C3 were reduced (Figure 2b,c down and Figure 2d right,#(hash) sign shows sig., The *p* values of WB were respectively 0.0082, <0.0001, 0.1998, and 0.0005; the *p* values of real‐time PCR were respectively <0.0001, <0.0001, 0.0127, and <0.0001). These results indicate that the autophagy markers were in general agreement with those observed by electron microscopy.

### 2.3 SIRT1 Induces Autophagy in HK2 Cells and Ameliorates Renal Tubular Epithelial Cell Fibrosis in Normoxia and Hypoxia Conditions

**Figure 3:**
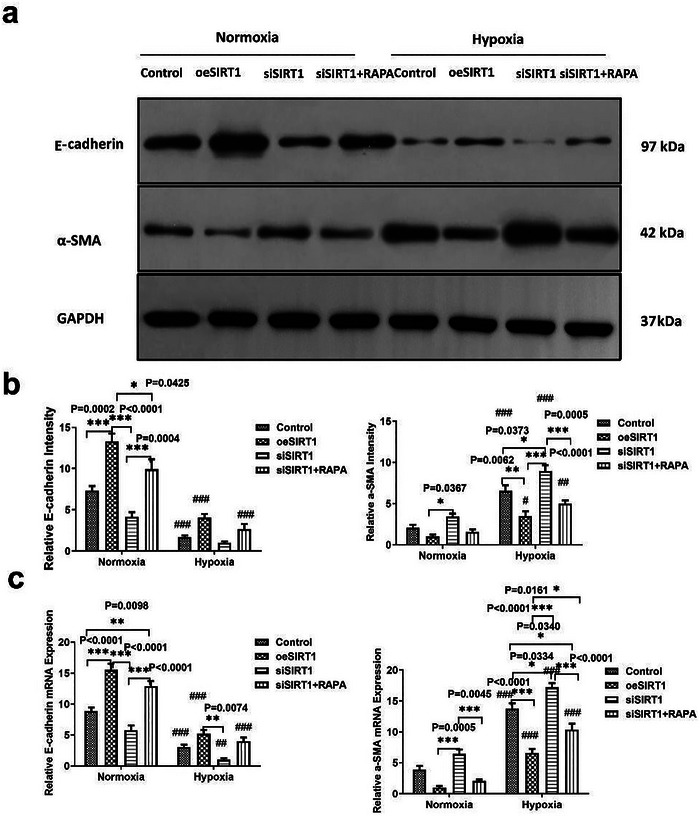
Characterization of the epithelial‐mesenchymal transition (EMT) in HK2 cells under normoxic and hypoxic conditions (*n* = 3). a) Western blot analysis was performed to detect the expression levels of E‐cadherin, α‐SMA, and GAPDH (used as a normalization gene) under normoxia and hypoxia conditions. b) The relative intensity of E‐cadherin and α‐SMA compared to the Control group was determined by western blot analysis software(The Discovery Series Quantity One 1‐D Analysis Software Version 4.6.8, Bio‐Rad, USA). (*n* = 3 samples per group, Control versus oeSIRT1 versus siSIRT1 versus SIRT1+RAPA: ^*^ *p* < 0.05, ^**^ *p* < 0.01, and ^***^ *p* < 0.001.Group under normoxia versus hypoxia: ^#^ *p* < 0.05, ^##^ *p* < 0.01, and ^###^ *p* < 0.001.) c) The relative mRNA expression levels of E‐cadherin and α‐SMA were evaluated using real‐time q‐PCR. (*n* = 3 samples per group, Control versus oeSIRT1 versus siSIRT1 versus SIRT1+RAPA: ^*^ *p* < 0.05, ^**^ *p* < 0.01, and ^***^ *p* < 0.001.Group under normoxia versus hypoxia: ^#^ *p* < 0.05, ^##^ *p* < 0.01, and ^###^ *p* < 0.001.) All experiments were repeated three times independently of each other. Note: Control versus oeSIRT1 versus siSIRT1 versus SIRT1+RAPA: ^*^ *p* < 0.05, ^**^ *p* < 0.01, and ^***^ *p* < 0.001. Group under normoxia versus hypoxia: ^#^ *p* < 0.05, ^##^ *p* < 0.01, and ^###^ *p* < 0.001. oeSIRT: overexpression of SIRT; siSIRTI: SIRTI knockdown using siRNA; siSIRTI+RAPA: knockdown of SIRT1 using siRNA with rapamycin‐induced autophagy.

**Figure 4:**
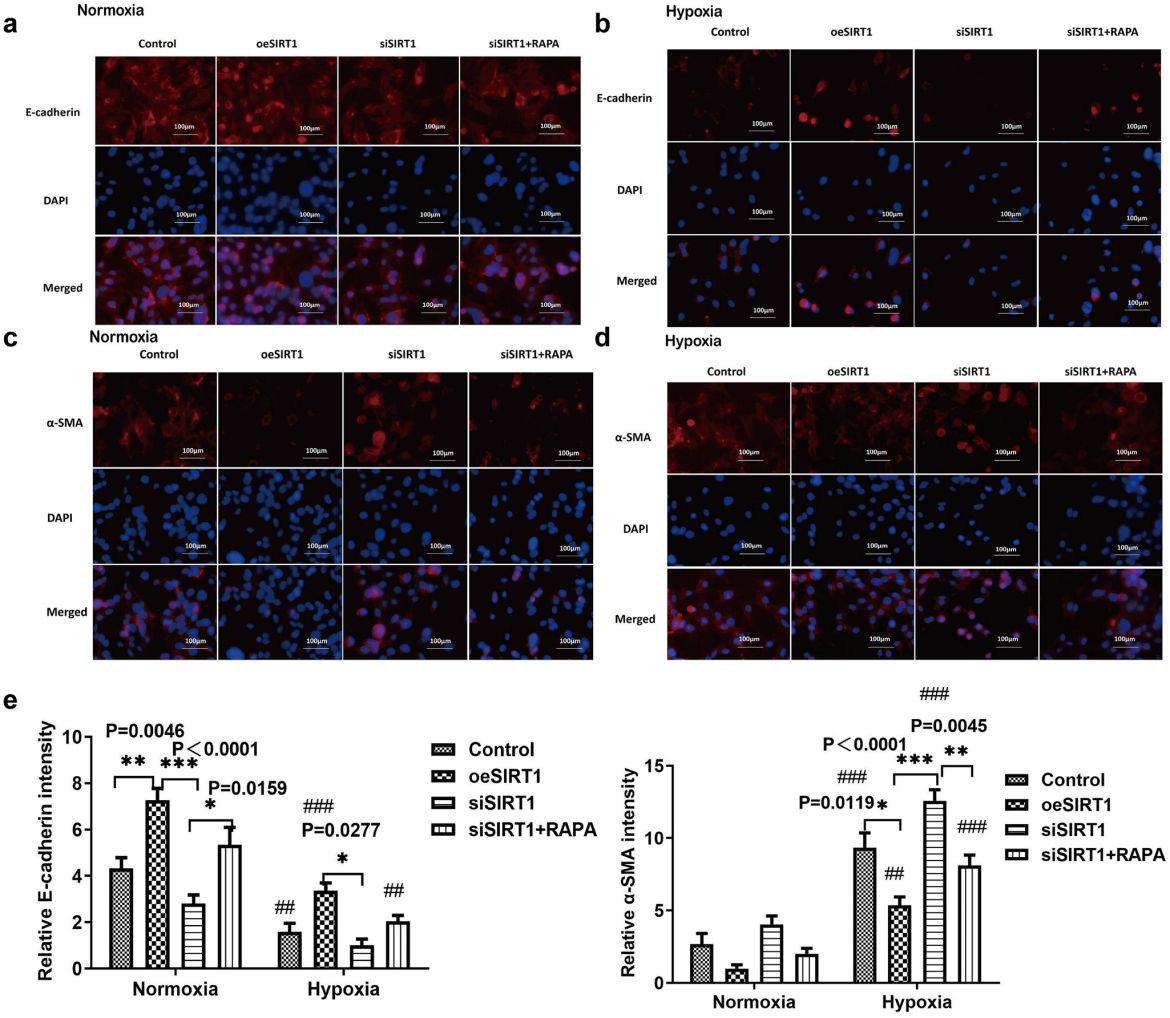
Semi‐quantification of E‐cadherin and α‐SMA in HK2 cells under normoxia and hypoxia conditions (*n* = 3). a) The analysis of E‐cadherin using Immunofluorescence (IF) assay in normoxia condition. b) The analysis of E‐cadherin using Immunofluorescence (IF) assay in hypoxia condition. c) The analysis of a‐SMA using Immunofluorescence (IF) assay in normoxia condition. d) The analysis of a‐SMA using Immunofluorescence (IF) assay in hypoxia condition. e) The relative fluorescence levels of E‐cadherin and α‐SMA were evaluated by Image J/fiji software. All experiments were repeated three times independently of each other. (*n* = 3 samples per group, Control versus oeSIRT1 versus siSIRT1 versus SIRT1+RAPA: ^*^ *p* < 0.05, ^**^ *p* < 0.01, and ^***^ *p* < 0.001.Group under normoxia versus hypoxia: ^#^ *p* < 0.05, ^##^ *p* < 0.01, and ^###^ *p* < 0.001.) Note: Control versus oeSIRT1 versus siSIRT1 versus SIRT1+RAPA: ^*^ *p* < 0.05, ^**^ *p* < 0.01, and ^***^ *p* < 0.001. Group under normoxia versus hypoxia: ^#^ *p* < 0.05, ^##^ *p* < 0.01, and ^###^ *p* < 0.001. oeSIRT: overexpression of SIRT; siSIRTI: SIRTI knockdown using siRNA; siSIRTI+RAPA: knockdown of SIRT1 using siRNA with rapamycin‐induced autophagy.

Furthermore, we evaluated E‐cadherin and α‐SMA expression using WB, qRT‐PCR, and IF and found that hypoxia promoted α‐SMA expression and inhibited E‐cadherin expression compared to normoxia (**Figures** **3** and **4**). These results showed that oeSIRT1 was increased in the E‐Cadherin group compared to the control group under normoxia conditions, and the expression of E‐Cadherin was significantly decreased in the siSIRT1 group compared to that of oeSIRT1 group, and after RAPA‐induced autophagy, the expression of expression α‐SMA was elevated (Figure 3a,b left,c left, and Figure 4e left, *star sign and *p* value are shown significantly on figures). In contrast to normoxia group, the relative expression level of E‐Cadherin was significantly reduced in four cell groups (Figure 3b left, c left, Figure 4e left, #hash sign shows sig., the *p* values of WB were respectively 0.0005, <0.0001, 0.0657, and <0.0001; The *p* values of real‐time PCR were respectively 0.0003, <0.0001, 0.0024, and <0.0001; The *p* values of IF were respectively 0.0087, 0.0003, 0.1439, and 0.0015).

**Figure 5:**
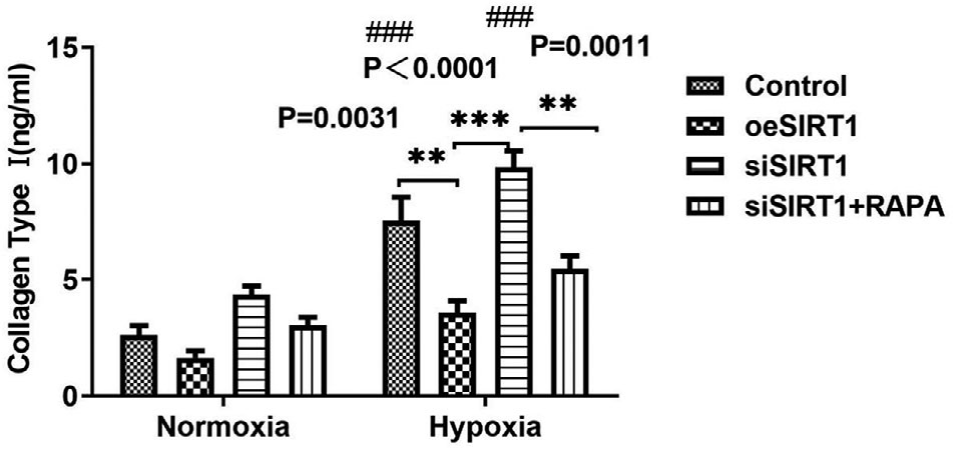
Quantification of collagen I concentration in HK2 cell culture supernatants. The concentration of collagen I in 8 groups(normaxia and hypoxic) was detected using ELISA (*n* = 3). All experiments were independently repeated thrice. (*n* = 3 samples per group, Control versus oeSIRT1 versus siSIRT1 versus SIRT1+RAPA: ^*^ *p* < 0.05, ^**^ *p* < 0.01, and ^***^ *p* < 0.001.Group under normoxia versus hypoxia: ^#^ *p* < 0.05, ^##^ *p* < 0.01, and ^###^ *p* < 0.001.) Note: Control versus oeSIRT1 versus siSIRT1 versus SIRT1+RAPA: ^*^ *p* < 0.05, ^**^ *p* < 0.01, and ^***^ *p* < 0.001. Group under normoxia versus hypoxia: ^#^ *p* < 0.05, ^##^ *p* < 0.01, and ^###^ *p* < 0.001. oeSIRT: overexpression of SIRT; siSIRTI: SIRTI knockdown using siRNA; siSIRTI+RAPA: knockdown of SIRT1 using siRNA with rapamycin‐induced autophagy.

However, the relative expression of α‐SMA was significantly elevated compared to that of each group under normoxia (Figure 3b right,c right, and Figure 4e right; #hash sign shows sig. The *p* values for WB were 0.0001, 0.0307, <0.0001, and 0.0021, respectively; the *p* values of real‐time PCR were <0.0001, 0.0004, <0.0001, and <0.0001, respectively; and the *p* values for IF were <0.0001, 0.0053, <0.0001, and 0.0002). We further quantified the concentration of collagen I in the cell culture supernatant, and the results of the four cell culture groups were evaluated under hypoxia (**Figure** **5**; *p* values are shown in figure). This suggests that fibrosis occurs in HK2 cells under hypoxic conditions. We further compared the levels of collagen I between the hypoxia and normoxia subgroups and found that the levels of collagen I in the control group(control) and the siSIRT1 group were significantly increased (Figure 5, #hash sign shows sig. The *p* values were respectively 0.0003, 0.3053, <0.0001, and 0.1160).

## 3 Discussion

In our research, we delved deeply into the crucial question of how hypoxia regulates HK2 cell autophagy via the SIRT1 gene and protein and triggers fibrosis in human renal tubular epithelial cells. Previous studies have reported that hypoxic conditions can induce a series of cellular stress responses in cancers and autoimmune diseases.^[^ ^15^, ^16^ ^]^ SIRT1, a significant deacetylase, plays a vital role in processes such as cell stress response, metabolism, and aging.^[^ ^17^ ^]^ Our experimental results demonstrated that, under hypoxic conditions, the expression level of SIRT1 was substantially altered and influenced the autophagy process of the HK2 cell line by FoxO1 and FoxO3 (Figures 1 and 2).

Through a succession of cellular and molecular detection methods, we determined that a hypoxic environment is capable of suppressing the activity and expression of SIRT1 protein, thus attenuating its regulatory impact on many downstream targets. Specifically, SIRT1 typically activates certain key transcription factors such as FoxO1 and FoxO3 through deacetylation, thereby facilitating the expression of genes related to cell autophagy.^[^ ^17^ ^]^ Nevertheless, under hypoxic conditions, the functionality of SIRT1 is compromised, resulting in an increase in the acetylation level of FoxO1, inhibition of its transcriptional activity, and, ultimately, a decline in the level of cell autophagy.^[^ ^10^ ^]^ Cell autophagy is an essential intracellular degradation and recycling mechanism crucial for maintaining intracellular homeostasis and responding to external stress.^[^ ^18^ ^]^ When the autophagy level drops, damaged proteins and organelles within the cells cannot be promptly cleared, and the accumulated damaged substances can trigger a series of cellular signaling pathways, eventually resulting in cell fibrosis.^[^ ^19^ ^]^

An important finding of our research is the elucidation of the mechanism of the SIRT1‐FoxO1‐FoxO3 signaling pathway and cell autophagy in improving renal tubular epithelial cell fibrosis. (Figures 1 and 2). Previous studies have reported that SIRT1 influences cell metabolism and stress responses by modulating FoxO1.^[^ ^10^, ^17^ ^]^ In our study, the pivotal role of the SIRT1‐FoxO1 signaling pathway in renal tubular epithelial cell fibrosis was further validated.

By implementing gene intervention and drug treatment in cells exposed to hypoxic conditions, we found that activating the SIRT1‐FoxO1‐FoxO3 signaling pathway could conspicuously elevate the level of cell autophagy, reduce intracellular damage accumulation, and effectively inhibit the progression of renal tubular epithelial cell fibrosis. Specifically, when the activity of SIRT1 protein is enhanced or restored, the acetylation level of FoxO1 or FoxO3 decreases, and its transcriptional activity increases, which subsequently promotes the expression of a series of genes related to cell autophagy, such as Beclin1 and LC3.^[^ ^20^ ^]^ Enhanced cell autophagy can clear damaged proteins and organelles, alleviate intracellular stress and damage, and improve fibrosis in the renal tubular epithelial cells.

Previous studies have achieved certain outcomes regarding the effects of the hypoxic environment on renal tubular epithelial cells and hepatocytes;^[^ ^21^, ^22^ ^]^ however, the specific mechanism of fibrosis caused by the regulation of cell autophagy by SIRT1 protein in the hypoxic environment remains poorly understood.

In contrast to previous studies, this study systematically probed the interrelationships between SIRT1 protein, FoxO1 transcription factor, and cell autophagy under hypoxic conditions and clarified their role mechanisms in renal tubular epithelial cell fibrosis. Additionally, we thoroughly verified the proposed mechanism and hypotheses through a series of experimental methods in vitro such as gene knockdown, overexpression, and drug intervention (RAPA). These findings not only enrich our understanding of the mechanism of renal tubular epithelial cell fibrosis in hypoxic environments but also provide a new target for future treatment strategies.

Despite obtaining certain results, this study still has some limitations and potential issues that warrant further discussion. This study primarily focused on exploring the impact of a hypoxic environment on renal tubular epithelial cell fibrosis at the cellular level, which has not yet been verified in animal models and/or patients in clinical settings. Although cellular experiments can provide certain mechanistic cues, animal models are better at simulating complex physiological environments and pathological processes in vivo. Furthermore, the regulatory mechanisms of the SIRT1‐FoxO1‐FoxO3 signaling pathway and cell autophagy in this study are not comprehensive or in‐depth. Although we found that SIRT1 protein regulates the activity of the FoxO1‐FoxO3 signal pathway through deacetylation to affect cell autophagy, there might be other undiscovered factors for regulation and other signaling pathways involved.

Additionally, the drug intervention and gene manipulation methods employed in this study may have limitations and safety concerns in clinical applications. Therefore, before converting these results into clinical treatment strategies, additional safety and efficacy evaluations are required.

The regulatory network of the SIRT1‐FoxO1‐FoxO3 signaling pathway and cell autophagy, searches for more key regulatory factors and signaling pathways, and provides a theoretical basis for the development of more effective therapeutic targets.

## 4 Conclusion 

This study demonstrates that the activation of SIRT1 can effectively attenuate renal epithelial cell fibrosis through the SIRT1‐FoxO1‐FoxO3‐Autophagy pathway. This discovery provides a novel theoretical foundation and potential therapeutic target for managing and preventing chronic kidney disease. Further investigations and clinical trials will contribute to the development of new treatment strategies in clinical practice.

## 5 Experimental Section

### Reagents and Instruments

DMEM culture medium (Gibco, USA), fetal bovine serum (Cat#10270‐106, Gibco, USA), trypsin (Hyclone USA), freezing vials, culture bottles, antibiotics (penicillin and streptomycin (100000 U/L each)) (Cat#KGY002, KeyGEN Biotech, China), 5% CO2 incubator (Cat#XD‐101, SANYO, Japan), clean bench (Cat#SW‐CJ‐1FD, Suzhou Purification, China), and an inverted phase‐contrast microscope (CAT#bx51, Olympus, Japan).

### Antibodies

Anti‐SIRT1 antibody (Cat# ab110304, Abcam, UK), Anti‐FOXO1A antibody(69 kDa) (cat# ab179450, Abcam, UK), Anti‐FOXO3A antibody(71 kDa) (Cat#ab109629, Abacm, UK), Anti‐Beclin 1 antibody(52 kDa) (cat# ab207612, Abacm, UK), Anti‐LC3B antibody(14, 16 kDa) (Cat# ab192890, Abacm, UK), Anti‐E Cadherin antibody(97 kDa) (Cat# ab231303, Abacm, UK), Anti‐alpha smooth muscle Actin antibody(42 kDa) (Cat# ab7817, Abacm, UK), Anti‐GAPDH antibody(37 kDa) (Cat# ab9485, Abacm, UK). SIRT1 overexpression vector and SIRT1 siRNA vector were synthesized by Shanghai GeneChem Biotech Ltd. (Shanghai GeneChem, Shanghai, China). Rapamycin was purchased from MedChemExpress (Cat#HY‐10219, MedChemExpress, China).

### Cell Culture

The human kidney cell line(HK2 cell, cat#1101HUM‐PUMC000160) was obtained from the Cell Resource Center, Peking Union Medical College (which is part of the National Science and Technology Infrastructure, National Biomedical Cell‐Line Resource, NSTI BMCR. http://cellresource.cn). The HK‐2 cells were cultured in DMEM medium supplemented with 10% fetal bovine serum (pH 7.2–7.4) at a temperature of 37 °C under a 5% CO2 atmosphere. The cells exhibited adherent growth and were enzymatically detached using 0.25% trypsin every 3 days. Cells in the normoxic group were incubated in a conventional incubator(Cat#XD‐101, SANYO, Japan) (21% O2, 74% N2, and 5% CO2) and cultured for 24 h. Cells in the hypoxic group were subjected to hypoxia training by introducing a gas mixture of 95% N2 and 5% CO2 into an O2 control incubator (Thermo HERAcell 150, USA) for 24 h.

### SIRT1 Over‐Expression and Knock‐Down

Cells cultured in plates were divided into four groups (control, oeSIRT1, siSIRT1, and siSIRT1_RARA) under normoxic (21% O2, 74% N2, and 5% CO2) and hypoxic conditions (95% N2 and 5% CO2).^[^ ^23^ ^]^ The control group comprised normal HK2 cell culture. The oeSIRT1 group was incubated with the SIRT1 overexpression vector. The siSIRT1 group was incubated with a SIRT1 siRNA vector to knock down SIRT1 expression. The siSIRT1+RAPA group was incubated with SIRT1 siRNA vector and Rapamycin (RAPA) to induce autophagy.^[^ ^24^ ^]^

### Western Blot (WB) Analysis

The cells were collected and lysed in a lysis buffer containing protease inhibitors(Cat#10 557, Yuanye Biotechnology, China). Protein concentrations were evaluated using a Pierce BCA Protein Assay Kit (Cat#23 225, Thermo Scientific, USA). Proteins (nearly 30 g) were immediately heated at 100 °C for 5 min, separated by SDS‐PAGE, and transferred to polyvinylidene fluoride membranes (cat#IPVH00010, Millipore, USA). The PVDF membranes were washed thrice for five minutes each with TBST. The membrane was blocked with 5% skimmed milk powder/TBST at room temperature for one hour. Primary antibodies, including the corresponding primary antibody diluted in a 1:500 antibody diluent and GAPDH antibody diluted in a 1:500 diluent, were added to the membrane and incubated overnight at 4 °C. After washing three times for 15 min each with TBST solution, Goat Anti‐Rabbit IgG H&L (HRP) (ab205718, Abcam, UK, Dilution: 1:5000) was added and incubated at room temperature for 45 min before washing with TBST solution three times for ten minutes each. The PVDF membrane was used to roll the film in a dark room, and with the scanning X‐ray film, an electronic image was obtained for semiquantitative analysis.

Image analysis software (Bio‐Rad, USA) was used to analyze the gray value of the destination stripe of each sample, with the objective of homogenizing the housekeeping gene GAPDH. Furthermore, the ratio of the normalized value of the reference stripe to the normalized value of each sample was set to 1 for the control and specimen to determine relative target protein expression.

### Realtime Fluorescent Quantitative Polymerase Chain Reaction (Real‐Time q‐PCR)

Total RNA was extracted from HK2 cell samples using the TRIzol Total RNA Extraction Kit (Cat#DP405‐02, Tiangen, China), according to the manufacturer's instructions. The quality and quantity of the extracted RNA were assessed using a spectrophotometer or a bioanalyzer. All primers used in this study were designed and synthesized by Invitrogen (**Table** **1**). For PCR amplification with SIRT1, FoxO1, FoxO3, Beclin1, LC3, E‐cadherin, α‐SMA, and GAPDH primers, 2 µL cDNA was used as a template. cDNA was generated using the PrimeScript RT Reagent Kit with gDNA Eraser (Cat# RR047B, TaKaRa, Japan) and SYBR Premix Ex Taq II ROX Plus. (TliRNaseH Plus)(Cat#, TakaRa, Japan). The Δ*C*_t_ value and amplification curve of each reaction were normalized, and the relative quantification 2‐ΔΔ*C*_t_ method was used to compare the expression variance of each gene.

**Table 1:** The primers of real‐time quantitative PCR.

Target gene	Sequence	
SIRT1	F	5′‐TCCTGGATAAGACCAAGTTTCTG‐3′
	R	5′‐ATAGATGTCAGCGATGGGTGTG‐3′
FOXO1	F	5′‐CTAAGGAGTTGCCGTTGTTGTACTGT‐3′
	R	5′‐AGACCCCACTTGAGATTCGTCA‐3′
FOXO3	F	5′‐ATTTGCTTTTGCCAAGAGTCAG‐3′
	R	5′‐GGTGTGCCTTCATATTCAAACC‐3′
Beclin 1	F	5′‐ GAGACAGCCAGGAGAAATCA‐3′
	R	5′‐ CCTGTGGATGACTGAGTACC‐3′
LC3B	F	5′‐GGGTGGCAGCTGACATGTTT‐3′
	R	5′‐GCCTTGAGCACCAGTTTGCT‐3′
E‐Cadherin	F	5′‐AATCCTGCTTGGGTATCAGG‐3′
	R	5′‐GAGACCCAGTCTCAGGGAAA‐3′
a‐SMA	F	5′‐TGTCATCTCGCTCTGGTACG‐3′
	R	5′‐AAATGACCCCTTCATCACCA‐3′
GAPDH	F	5′‐AATCCTGAGCAAAGCTGAGAAC‐3′
	R	5′‐CGTACAAAACCAATCACGAGAA‐3′

### Immunofluorescence (IF) Assay

E‐cadherin and α‐SMA levels were analyzed as described previously.^[^ ^9^ ^]^ Briefly, cells on slides were fixed, permeabilized, and stained with anti‐E‐cadherin antibody (cat#ab231303, Abcam, UK, Dilution:1:200)or anti‐alpha smooth muscle actin antibody (a‐SMA)(cat# ab7817, Abcam, UK, Dilution: 1:500)at room temperature for 2 h. After removing the excess primary antibodies with 1×PBS, the cells were incubated with Goat Anti‐Rabbit IgG H&L (Alexa Fluor 594) pre‐adsorbed (Cat#ab150084, Abcam, UK, 1:1000) for 2 h. Nuclear DNA was labeled with a DAPI solution (Cat#MBD0015, Sigma‐Aldrich, USA). Immunostaining image characteristics were assessed independently using the ImageJ software and compared with those of the control group.

### Transmission Electron Microscopy

HK‐2 cells, consisting of renal tubular epithelial cells from the different treatment groups, were collected. The cells were centrifuged to remove the supernatant, washed twice with 1×PBS, and fixed using 2.5% glutaraldehyde after cluster formation. All cells in the group were washed with 0.1 m phosphate buffer three times, followed by fixation in 1% osmic acid (Pelco, USA) at 4 °C for 3 h. Subsequently, they were subjected to additional buffer washes, ethanol dehydration, substitution with epoxy propane, and embedding in saturation using the Spurr Low‐Viscosity Embedding Kit (Cat# EM0300, Merck, USA). Subsequently, the embedded blocks from different groups were sectioned using an ultrathin microtome. The resulting ultrathin sections were 70 nm thick and were stained with uranyl acetate (CAT# 02624‐AB, Spi‐Chem, USA) and lead citrate (CAT# 02617‐AB, Spi‐Chem, USA) before observation and photography under a transmission electron microscope.

### Enzyme‐Linked Immunosorbent Assay (ELISA)

After the removal of impurities, the supernatant was carefully transferred to a new tube and stored at −80 °C for further analysis. The concentration of collagen I in the samples was determined using a human collagen I ELISA kit (Cat#CSB‐E08082 h, Cusobio, China), following the manufacturer's instructions. This involved preparing standards, controls, and samples in duplicate wells, incubating with detection reagents A and B successively, washing the plate thoroughly, adding a substrate solution for color development, and finally stopping the reaction with the stop solution. The absorbance of each well was measured at 450 nm using a microplate reader within 15 min of adding the stop solution. The concentration of collagen I in each sample was calculated based on a standard curve generated from optical density readings of the standards. All procedures were performed according to established protocols to ensure accurate and reliable results for further analysis.

### Statistics

All statistical analyses were conducted with GraphPad Prism 9 software. All experiments were repeated independently at three times in this study, with three biological replicates for each group. Data are expressed as mean ± standard deviation, with results presented either from representative experiments or pooled across all study. For cell culture experiments, immunofluorescence, real‐time ‐qPCR, and Western Blot were quantified using averaged values (calculated from 3 per test, constituting one biological replicate Statistical comparisons were performed on the averaged dataset. Between group comparisons were assessed using two‐tailed Student's *t*‐tests for dual‐group experiments. Multi‐group comparisons utilized either *one‐way or two‐way ANOVA* followed by Tukey's post‐hoc test. The statistical significance threshold was set at α = 0.05 for all analyses. Multi‐factor ANOVA was performed to compare intergroup differences, with the significance level set at α = 0.05. Statistical significance was defined as *p* < 0.05, while ​^**^ *p* < 0.01, and ​^*^ *p* < 0.001​ were interpreted as highly significant and extremely highly significant, respectively. Within‐group comparisons (Control vs oeSIRT1 vs siSIRT1 vs SIRT1+RAPA) are denoted by asterisks (*), and between‐group comparisons (normoxia vs hypoxia) by hash symbols (#).

## Conflict of Interest

The authors declare no conflict of interest.
